# Prevalence of gestational diabetes in Eastern Democratic Republic of Congo

**DOI:** 10.1186/s12884-022-04970-y

**Published:** 2022-08-16

**Authors:** Rogatien Mwandjalulu Kisindja, Pierrot Lundimu Tugirimana, Mitangala Ndeba Prudence, Katenga Bosunga, Jean-Jeannot Juakali Sihalikyolo, Prosper Kalenga Muenze Kayamba, Albert Mwembo Tambwe-A-Nkoy

**Affiliations:** 1 Department of Obstetrics & Gynecology, La Sapientia Catholic University (UCS)/Goma, Goma, Congo; 2 Department of Internal Medicine, University of Goma (UNIGOM), Goma, Congo; 3 Official University of Ruwenzori (UOR) Public Health, Butembo, Congo; 4 Department of Gyneco-Obstetrics, University of Kisangani (UNIKIS), Kisangani, Congo; 5 Department, of gyneco-obstetrics & public health, University of Lubumbashi (UNILU), Lubumbashi, Congo

**Keywords:** Gestational diabetes, Eastern DRC, Prevalence

## Abstract

**Objective:**

To determine the prevalence of gestational diabetes and associated risk factors in the eastern region of the Democratic Republic of Congo (DRC).

**Methods:**

A cross-sectional study was conducted in Goma city, Idjwi, Ngungu and Rutshuru districts between April 2019 and February 2021. Pregnant women between 24–28 weeks of amenorrhea who consented to participate in the study were included. Blood sugar, anthropometric parameters and obstetrical and family history were studied. Gestational diabetes was defined as blood glucose level between 92 and 125 mg/dL.

**Results:**

The overall prevalence was 21.2% (*n* = 391) and was higher in Rutshuru [27.2% (*n* = 92)] and Goma [26.9% (*n* = 134)] compared to Ngungu [10.0% (*n* = 110)] (*p* = 0.005). An increased risk was associated with a history of a newborn weighing ≥ 4000 g [OR 2.4 95% CI (1.3 – 4.4)] or family diabetes [OR 2.9 95% CI (2.0 – 4.9)]. Median age in the pathological group was not different from that with normal blood glucose [25.0 (16.0 – 44.0) Vs 26.0 (16.0 – 44.0)] (*p* = 0.67). The prevalence tended to increase for pregnant women with a mid-upper arm circumference ≥ 280 mm [28.1% (*n* = 57)] Vs [19.3% (*n* = 322)] if < 280 mm, [OR (95% CI)] [1.5 (0.9—2.3)] (*p* = 0.13).

**Conclusion:**

Gestational diabetes was found in one out of five pregnant women regardless their age. A history of macrosomia birth and diabetes in the family were the main risk factors.

**Supplementary Information:**

The online version contains supplementary material available at 10.1186/s12884-022-04970-y.

## Introduction

The World Health Organization (WHO) defines gestational diabetes (GDM) as an intolerance to glucose marked by hyperglycemia of variable severity, which appeared or was discovered for the first time during pregnancy, regardless of its evolution after childbirth [1].

Pregnancy follow-up, whatever the level of the health facility, should methodically make it possible to detect glucose intolerance according to the required criteria without missing borderline situations, because of epidemiological presumptions [2], even if it there is some controversy over the standard test for screening [3].

Moreover, the discovery of GDM portends a recurrence of 30–84% and the risk of type 2 diabetes is multiplied by 7 [1]. This risk can reach 60% within 5 to 15 years after delivery [3].

GDM is a public health problem. Worldwide, its frequency continues to grow [4]. Globally the frequency of undiagnosed diabetes before pregnancy and of GDM are estimated between 10 to 25% of pregnancies [5]. This frequency may vary according to regions, countries or continents. In 2021 it was reported 13% in Africa up to 25.9 in South-Eastern Asia and the worldwide average was 16.7% [6].

In Europe, the prevalence was 22.3% varying between 13 and 31.5% in 2016 [1]. In Asia, figures of 11.5% reported in 2018 [6]. While in mainland China a prevalence of 14.8% was observed [7]. In 2017 it was 17.8% in Canada [8].

In Africa, prevalence was 13% in Arusha/Tanzania in 2019 [9], 33.1% in Dakar/Senegal in 2015 [10] and 38.8% in Abidjan/Ivory Coast in 2008 [11].

In the Democratic Republic of Congo (DRC), the prevalence of GDM remains imprecise and sectoral. Frequencies of 44.0% have been described in Bukavu, south Kivu [11] and 3.9 to 5.2% in Kinshasa [12].

The objective of the present study was to determine the prevalence of GDM and its associated risk factors in Goma, Idjwi;. Ngungu and Rutshuru.

## Materials and methods

### Study design and site

This cross-sectional and multicenter study was conducted from 04/25/2019 to 02/02/2021 in eastern DRC in sites chosen for their geographical characteristics of altitude and temperature. These included the Mapendo health center in Goma, the Ngungu reference health center and the Rutshuru health center in North Kivu province and the Bunyakiri health center on the island of Idjwi in the province of South Kivu.

### Study population

This study involved pregnant women who had followed the prenatal consultation in the sites and during the study period and who had consented to participate and whose term of pregnancy was between 24 and 28 weeks of amenorrhea, who had observed a fasting period of at least 8 h and who had no known chronic disease and were not on corticosteroid therapy.

### Sample size and sampling

The prevalence of type II diabetes, generally accepted as the closest reflection of the probability of GDM because it is parallel to it [13]. The prevalence of type II diabetes in the DRC being 18.4% [14], a proportion of 20% was considered as a reference. We then adjusted a 10% margin for non-respondents.

Thus, for a prevalence of 20%, taking a confidence level of 95% and a margin of error of 5%, the sample size should be n = t^2^ × p × (1-p) / m^2^ i.e. 1 0.96^2^ × 0.2 × 0.8 / 0.05^2^ = 245.8. Considering a margin of 10% for non-responders: 245.8 + 10% (24.58) = 270.3. The minimum number for the purposes of the study was therefore 270 subjects. But considering the diversity of the DRC and the sectoral nature of the data available on diabetes, we have, for reasons of simplicity and to have enough subjects, used a sampling proportional to the cases expected on the different sites. The number of participants per health center was calculated based on the relative contribution of each center to the total number of women who were followed for antenatal care (ANC) per site during the six months preceding data collection. The total number of pregnant women who attended ANC during that period was 6895. The relative frequency of ANC per health facility was used to calculate the following indexes: 0.345 for Goma; 0.140 for Idjwi;0.280 for Ngungu and 0.235 for Rutshuru. For convenience and in order to get at least 50 participants per site, the the indexed proportion was multiplied by 1,45 to get a total number of 392 pregnant.

### Data collection 

Staff at the various sites has been trained to standardize data collection. Data collection was done on the basis of a standard survey form. Informed consent was systematically requested for the collection of personal data, guaranteeing anonymity in the processing of data in order to respect the principles of confidentiality and human dignity. The socio-anthropometric data of the pregnant women, the gestation, and the parity, the history of diabetes in the family and of the child of weight ≥ 4000 g at birth were collected on a data collection sheet. Because of lack of both standard laboratory equipment and qualified laboratory staff in these remote and rural health facilities, only capillary blood glucose level determination was technically possible.

Pregnant women with a higher blood sugar level ≥ 92 mg/dl received lifestyle and dietary advice and were reviewed two weeks later.

### Laboratory methods

Blood glucose was measured using a commercial glucose meter (SAFE-ACCU Blood Glu-cose Monitoring System, Changsha Sinocare, Inc., P.R.C.).. [15]. The accuracy and precision of the glucometer was evaluated making use of quality control materials (Acusera, Randox Laboratory Ltd, Crumlin, UK). The quality control was performed at the opening of each lot of 50 test strips. GDM was defined as blood sugar between 92–125 mg/dl and normal blood sugar as between 60 and 91.9 mg/dl.

### Statistical analyzes

The data collected was encoded in Excel and analyzed using SPSS version 23 software. Capillary blood glucose had been studied according to socio-anthropometric parameters, gravidity and parity, history of diabetes in the family and child weight ≥ 4000 g at birth. The synthesis of the variables, whose distribution in the sample was asymmetrical, was made in terms of median accompanied by its range of variation or after transformation into categories, expressed in proportion (%) like the qualitative variables. Pearson's Chi2 and/or Fisher's exact test were used to compare the proportions. The Wilcoxon-Mann–Whitney test was applied for the comparison of the medians. The significance level was set at 0.05.

## Results

### Of 2687 pregnant women undergoing antenatal consultation (ANC) for the first visit during the sequential data collection periods, 392 had been enrolled, a proportion of 14.6%

#### Sociodemographic and gestational characteristics

The socio-demographic, nutritional and obstetrical parameters of the pregnant women included in the study are summarized in Table 1. Of a sample of 392 pregnant women included in the study, 135 came from Goma, 110 from Ngungu and 92 from Rutshuru in the North Province Kivu and 55 from Idjwi in the province of South Kivu. The median age (minimum – maximum) was 25.50 (16 -44) years. The median gravidity was 4 (1–12) and the median parity was 2 (0–11). Just over 45% of pregnant women were overweight. The frequency of overweight pregnant women was high in Goma [71.1% (*n *= 135)] and Rutshuru [40.4% (*n* = 89)] compared to Idjwi [10.9% (*n* = 55)] (*p*˂ 0.001). This frequency in Ngungu was 35.5% (*n* = 110). About 15.0% of pregnant women had an arm circumference ≥ 280 mm.

**Table 1:** Socio-demographic nutritional and obstetrical parameters of pregnant women included in the study

**Setting**	**Median (Min – Max)**	**Proportion 95%CI**
Age in years (*n*=392)	25.50 (16 – 44)	
Gestation (*n* = 392)	4 (1 – 12)	
Parity (*n*=392)	2 (0 – 11)	
Weight in Kg (*n* = 389)	64 (46 – 95)	
Arm circumference in mm (*n* = 379)		
< 230 mm		9,8(6,8 – 12,8)
230 - 250 mm		9,8(6,8 – 12,8)
˃250 mm		64, 1(59,3 – 68,9)
Body Mass Index in Kg /m² (*n* = 385)		
< 18,5		0,5(0,2 – 1,2)
18,5 - < 25		54,3(49,3 – 59,3)
25 - < 30		36,1(31,3 – 40,9)
≥ 30		9,1(6,2 – 12,0)
History of macrosomia (*n*=351) Yes		4.6(2,4 – 6,8)
Family history of diabetes (*n*=383)		4.7(2,3 – 6,3)
Family history of chronic disease (*n*=66) Yes		7.6(1,2 – 14,0)
Fasting duration in hours (*n* = 388)	12.3 (9.0 – 25.1)	

### Prevalence of GD

The overall prevalence of GDM was 21.2% (n = 391). One pregnant woman had a blood sugar level of ≥ 126 mg/dL.

There was no statistically significant difference in GDM prevalence by age, gravidity, parity, mid-upper arm circumference ( MUAC), and body mass index (Table 2).

**Table 2:** Blood sugar levels according to the different parameters of pregnant women

Setting	92—125 mg/dL	< 92 mg/dL	p
Age in years	*n* = 83	*n* = 307	
Median (Min—Max)	25.0 (16.0 – 44.0)	26.0 (16.0 – 44.0)	0.67
	*n* = 83	*n* = 307	
GravidityMedian (Min—Max)	3 (1 – 11)	4 (1 – 12)	0.80
Parity	*n* = 83	*n* = 307	
Median (Min—Max)	2 (0 – 10)	3 (0 – 11)	0.64
Arm circumference in mm	*n* = 77	*n* = 300	
Median (Min—Max)	260 (208–330)	260 (160 – 352)	0.38
Weight in Kg	*n* = 82	*n* = 305	
Median (Min—Max)	63 (47 – 95)	64 (46 – 90)	0.49
Fasting duration in hours	*n* = 83	*n* = 303	
Median (Min—Max)	12.3 (11.0 – 14.1)	12.4 (9.0 – 25.1)	0.26

Although GDM seemed more frequent in pregnant women with arm circumference ≥ 280 mm [28.1% (*n* = 57)] compared to those with arm circumference < 280 mm [19.3% (*n* = 322)], the difference was not statistically significant [OR (95% CI)] [1.5 (0.9, 2.3)] (*p* = 0.13). GDM prevalence was significantly higher in Rutshuru [27.2% (*n* = 92)] and Goma [26.9% (*n* = 134)] compared to Ngungu [10.0% (*n *= 110)] (*p* = 0.005). In Idjwi, it was 20.0% (*n* = 55). The high prevalence of GDM was associated with a history of diabetes in the family or with a child with a birth weight of more than 4000 g (Table 3).

**Table 3:** Blood sugar levels according to history of diabetes and macrosomia of pregnant women

Setting	% Glucose 92—125 mg/dL	OR (95% CI)	p
Family Diabetes history			
Yes (*n* = 17)	58.8	3.1 (2.0 – 4.9) 26.0	
No (*n* = 364)	19.0	1	< 0.001
Macrosomia history(Min—Max)			
Yes (*n* = 16)	43,8	2.4 (1.3 – 4.4)	
No (*n* = 333)	18.3	1	0.021

## Discussion

The study was conducted in 4 sites in the two provinces of North and South Kivu in the eastern part of the DRC in order to determine the prevalence of GDM and the associated factors. The overall prevalence of GDM was 21.2%. Family history of macrosomia and diabetes were significantly associated with high prevalence of GDM. The prevalence observed in our study is higher than that observed in Kinshasa by Tandu-Umba NFB & al. [14], to those reported by Msollo in Arusha in Tanzania [8] and to those reported in Asian studies [6]. It is lower than those observed in Dakar in Senegal [9], in Abidjan in Ivory Coast [10] and in Bukavu in the DRC [14]. The fact that in Dakar and Abidjan, the studies were carried out in a hospital environment where all the cases that could present a risk are normally taken care of and that the screening included, as in Bukavu, induced oral hyperglycemia, could explain the difference with the results observed in this study. The frequency of GDM observed in this study is close to the average European median prevalence reported by N. Pirson & collaborators [1].

The observation that pregnant women with GDM tended to be younger is in line with the observation of Orru MI et al. who, speaking of gestational pre-diabetes, had observed that relatively younger pregnant women were also affected and this should not exclude them from screening for GDM [2]. The age level of the pregnant women in this study is comparable to that done by Kodjo Agbeko and by Leye A. who respectively observed mean ages of 30.84 ± 4.17 years [16] and 29.8 ± 6.2 years [9]. The observation on this level of young age differs from those of most authors such as Tandu-Umba in Kinshasa [14], Tieu J [17] and Diane Farrar [4], who had observed that advanced age was a risk factor for eligibility for selective GDM screening. There is little consensus on age as a criterion for selective screening of GDM as recommended by certain groups of experts cited by Mirghani Dirar [18], contrary to the observation of Herath HM M who found GDM in increasingly young pregnant women in Sri Lanka [3]. This could suggest further exploration of this parameter as a criterion for selective screening. Other factors including geographical and climatological conditions could be determinants [19, 20] and parity did not show any relationship with GDM. This differs from the observation of Leye A who found that multiparity was significantly associated with GDM [10]. Nutritional factors assessed by MUAC [21] did not show a significant relationship with GDM. This is contrary to the result of Msollo & al. who had observed that arm circumference ≥ 280 mm was associated with hyperglycemia during pregnancy [9]. Although the weight before conception was not known in this study, the literature notes that overweight and obesity before conception was accompanied by a greater risk of having GDM, as retained by the various groups of experts [8]. For Amy Shah, BMI ˃ 25 would not be a good indicator for selective screening. Its sensitivity would vary according to the ethnic groups [22]. For Da Yao, better than BMI, maternal central obesity during the first two trimesters was more associated with the risk of GDM [23]. As for Leye A, he also had not found obesity as a risk factor for GDM [10].

The observation made in this study that a history of macrosomic delivery was significantly associated with GDM corroborates with the various authors such as Tieu J &all. [17]. For certain groups of experts quoted by Mirghani Dirar A. [8] the history of an new-born macrosomic infant is a determining factor of GDM although for them, macrosomia corresponded to a weight ≥ 4500 g.

Family history of diabetes was significantly associated with GDM; this corroborates with the observation made by Tieu J [17] and the groups of experts quoted by Mirghani Dirar A. [8].

This study has some limitations. The first limitation relates to the sites where the study was carried out. The localities selected may not reflect the reality of the entire population of the provinces of North and South Kivu in the eastern part of the DRC. Disparities could exist from one locality to another depending on certain parameters, in particular the diversity of habits of the Congolese population. The second limitation is linked to the measurement of blood glucose levels. A previous study by S. D. Pastakia et al. [24] has shown that missed cases may reach one out of three cases. It showed that the venous fasting blood glucose measurement could diagnose only 12 out of 18 GDM detected by the oral glucose tolerance (OGTT) test criteria [24]. And finally, the lack of OGTT is the third limitation in this study. However the fasting capillary blood glucose remains a GDM screening strategy in low resources countries [25]. Despite these limitations, this study has merits. To our knowledge, it is one of the few studies conducted in this region to estimate the extent of GDM.

## Conclusion

GDM is an under-explored public health problem in eastern DRC. A little more than 1 in 5 pregnant women are affected regardless of their age. The history of a newborn weighing ≥ 4000 g and diabetes in the family is an important risk factor for GDM. Given the sectoral nature of data on diabetes in the DRC, highly powered studies may prove necessary in order to better estimate the extent of this problem.

### What is known?

GDM is a public health problem and its frequency continues to grow worldwide. A history of diabetes in the family, delivery of a macrosomic newborn, obesity before conception are distinctly risk factors for GDM.

### Contribution of this study

This study specifies the prevalence of GDM, demonstrates that the pathology concerns pregnant women regardless of their age and supports that young pregnant women should not be excluded from selective screening. GDM is a pathology encountered in our environment with a different frequency depending on the study sites.

## Supplementary Information

**Additional file 1.**

## Data Availability

The data and materials are in synapse storage: https://www.synapse.org/#!Synapse:syn29218130. Alternatively, reasonable data request can be made to the principal investigator.
